# Patellar Tendon Thermographic Reference Values in Healthy People: A Systematic Review

**DOI:** 10.3390/muscles3040030

**Published:** 2024-10-18

**Authors:** Roberto Mevi, Alessio Cabizosu

**Affiliations:** THERMHESC Group, Chair of Ribera Hospital de Molina San Antonio, Catholic University of Murcia (UCAM), 30107 Murcia, Spain; rmevi@alu.ucam.edu

**Keywords:** reference values, thermography, patellar tendon, health

## Abstract

Introduction: The physiological response of the tendon structure has been the subject of several studies using clinical thermography, although the lack of normal values limits clinical practice despite being, according to several authors, an optimal diagnostic tool for the study and therapeutic monitoring of soft tissues. To this end, the aim of this systematic review was to explore all the scientific information on thermography and reference values in the patellar tendon. Method: A search was carried out in different health science databases using the MeSH terms “Health”, “Patellar Ligament”, and “Thermography” with their synonyms in free terms to collect the studies to be included in the systematic review. This review was conducted according with PRISMA guidelines and includes all of the literature up to 19 December 2023. All studies that were in accordance with the PICOS strategy in title and abstract were included in this review, while studies that performed the baseline thermographic test in an altered physiological state were excluded after reading the full text. The TISEM scale was used to assess the quality of the studies; the risk of bias was assessed with the QUADAS-2 scale. Result: A total of 6 articles was selected (n = 148); these were analyzed for quality and found to be highly heterogenous; the risk of bias was low in most domains of the QUADAS-2 scale. Discussion and Conclusion: There are several variabilities that can induce changes in the thermoregulation process distorting the thermograms. Looking deeper into the evidence behind each item, we can deduce the need to follow the TISEM protocol as closely as possible for an accurate response. The heterogeneity in the way the results were provided and the low quality in some of the studies did not allow for a reference of thermographic values to be obtained.

## 1. Introduction

In recent years, the demand for clinical care due to pain or injuries in tendon tissues has increased notably, especially in the patellar tendon, and it has been determined that a good diagnosis is essential in the prevention, treatment, and follow-up of this pathology [1,2,3]. It is known that tendinopathies in are due to muscular deficiency many instances, since due to the lack of strength and repetitive movement, the tendon suffers excess stress, generating an inflammatory process as a defense mechanism [4,5]. Currently, the diagnosis of tendon pathologies is based both on the clinical history and clinical examination of the patient and the analysis of imaging tests, such as ultrasound and magnetic resonance imaging (MRI) [6] because these tests provide objective and reliable quantitative information regarding the tendon status in healthy patients or the degree of injury in affected patients, providing very important structural information. However, some authors describe a discrepancy between the images and the symptoms reported by the patients [7]; therefore, some authors have recently proposed to implement a new tool for the assessment of this tissue, addressing not only structural but also metabolic aspects.

For example, it is known by other authors that the diagnostic perspective provided by these techniques shows that the tendon volume and the organization of fiber distribution are related to the appearance of inflammatory processes characteristic of tendinopathies; however, it is not possible to associate this morphological and structural change with the clinical processes or symptoms of the patients [8,9]. This could be due on the one hand to the limitations of these diagnostic techniques and on the other hand to the subjective interpretation of these images by the experts, since both in the case of ultrasound and in the case of magnetic resonance, in spite of observing a high sensitivity in the clinical morphological analysis [10], this may not correlate with pain or mechanical function [11].

Moreover, since these two diagnostic techniques do not provide qualitative information about the results of the treatment performed or the metabolic and evolutionary state of the lesion, once the medical diagnosis has been established, the clinician relies on exclusively subjective data for the treatment of the functional diagnosis, without the possibility of re-evaluating the effects of the techniques in an objective and reliable manner in the short term. In this sense, thermography in recent studies has been used as a complementary tool to the main diagnostic techniques, to clarify aspects that may go unnoticed with other diagnostic elements. The term clinical thermography refers to a medical diagnostic technique capable of capturing the infrared radiation emitted by all bodies and surfaces with a temperature above absolute zero due to the molecular agitation intrinsic to matter [12]. In relation to tendinopathies, a recent systemic review by Dias de Lacerda et al. [13] shows that this imaging technique has been shown to be reliable in diagnostic accuracy, with high specificity, especially in the shoulder and elbow regions.

In health sciences, this technique has been implemented in several areas such as oncology, neurology, and dermatology, although it has recently found great activity in sports medicine, analyzing the metabolic muscle activity of athletes in different disciplines [14]. Reliability and reproducibility studies have given thermography an important role in the field of health sciences, something that had not been planned, as its first appearances in the scientific literature were related to the military and construction [15].

Although the number of studies related to thermography in the health sciences has increased in recent decades, it should be noted that most of these studies have been carried out on pathological samples, leaving aside the importance of determining normal values. This could be due, according to some authors, to the difficulty of generating homogeneous data of thermographic normality due to the great variability of the metabolic processes that can influence the analysis of thermographic images, [16] although recently, this need is being solved since several studies have been published on the use of thermography as a tool to determine these reference values in hands [17], in upper and lower limbs by points [18,19], and in knee prostheses for the diagnosis of infections [20].

However, in relation to the patellar tendon, the absence in the literature of these thermographic reference values for the patellar tendon in healthy populations means that the results reported by the images are interpreted by different professionals in a biased and, therefore, limited way.

Therefore, the objective of this systematic review was to explore all the scientific information on thermography and reference values in the patellar tendon, with the aim of identifying discrepancies in the literature and clarifying the most current knowledge. A review of this type is necessary because, on the one hand, there is no previous work of this type and, on the other hand, research in this field has generated new knowledge that needs to be unified and analyzed.

## 2. Method

### 2.1. Study Design and Documentary Resources

A systematic literature review was conducted using electronic queries following the Preferred Reporting Items for Systematic Reviews and Meta-Analysis (PRISMA) standard [21]. In order to generate the most exhaustive and reliable information possible, different computerized searches were carried out on multiple databases and editorial platforms: MEDLINE using Pubmed, LILACS and IBECS in the Virtual Health Library (VHL), CENTRAL through Cochrane Library, in “WoS Core Collection” and SciELO through “Web of Science” and CINAHL complete, and SPORTDiscuss in the EBSCO host database. The last search was carried out on 3 December 2023.

### 2.2. Search Strategy

The specific PICOS strategy [22] terms “Healthy”, “Thermography”, and “patellar tendon” were used to perform the search lines, using the combination of the logical Boolean operators “AND” and “OR” between the English thesaurus terms, their synonyms in the form of free terms, and truncated terms. The terms used were Health OR Healthy OR Normal* AND Thermograph* OR “Temperature mapping” OR “Infrared body temperature” OR “Thermal imaging” OR “DITI” OR “Infrared thermography” AND “Patellar ligament” OR “Patellar tendons” OR “Ligamentum patellae”.

### 2.3. Study Selection Process

All the literature on patellar tendon thermographic values in a healthy population was included in the study according to the PICOS strategy [22]. In order to collect as exhaustive a set of information as possible, no exclusion criteria were established regarding date of publication or language of the articles. Furthermore, we did not discriminate by journal or manuscript author. Studies in which the sample was under physiological stress prior to measurement, studies that could not be found in full text, where it was not possible to locate the abstract or where the results were not fully explained, letters to the editor, and/or conference presentations were excluded. Within the recruited studies, duplicates were discarded through the artificial intelligence (AI) “Rayyan.ai” [23]. The screening process of the studies was carried out individually in two phases by 2 blinded authors. In the first phase, it was checked whether the terms of the PICOS strategy [22] were present in the title or abstract, while in the second phase it was checked whether the extrapolated data were available in the full text. The 2 authors participated in the selection and analysis of the included articles, and when there was doubt or debate, it was solved between them. After obtaining the final results, all references were extracted, and to systematize the studies, they were imported into Zotero (5.0.64, Zotero, Fairfax, VA, USA). After organizing the bibliography, a further fan search was performed to ensure complete and exhaustive information.

### 2.4. Data Extraction

Data extraction was performed in a blinded manner and compared between the 2 authors in order to select relevant and appropriate studies for this review. Data on study characteristics (author, year of publication, study design, country of sample, country of publication, and journal), participant characteristics (sample size, age, and sex), characteristics of the thermographic intervention (TISEM scale) [24], and on the characteristics of the thermographic response obtained (mean temperature at baseline and type of thermograph used) were selected.

### 2.5. Risk of Bias and Evidence Quality

The tool used to assess the risk of bias was the Quality Assessment Diagnostic Accuracy Studies-2 (QUADAS-2). QUADAS-2 consists of 4 domains: patient selection, index test, reference test and flow and timing. Of the 4 domains, risk of bias is assessed and the first three are also assessed for applicability [25]. The quality of the evidence was assessed through the 15 items of the Thermography Imaging in Sport and Exercise Medicine (TISEM) consensus that evaluates different factors on the sample information: the environment where the thermographic imaging was performed, the preferences of the cameras used, the analysis, and the reporting of the data [24]. This scale has been used previously by other authors to assess the methodological quality of thermographic studies [26]. This tool provides 15 items, evaluable with “Yes”, “No”, and “Unclear” depending on whether what the item describes is present in the study (e.g., description of reported temperature and humidity with standard deviation, description of chamber emissivity settings, etc.) [22].

## 3. Results

### 3.1. Process of Identification and Selection of Studies

**Figure 1:**
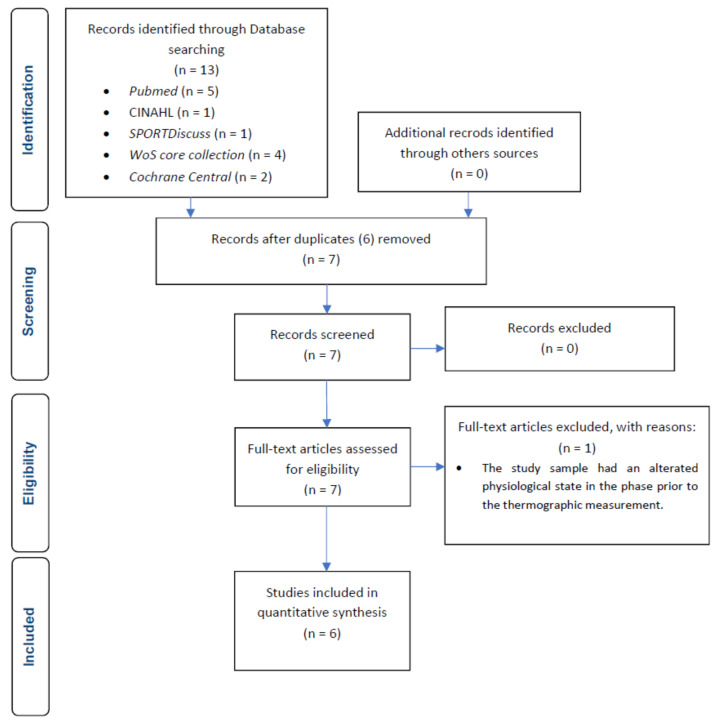
Flow chart of studies selection process.

A total of 13 records were recruited for this review after the first search process. As can be seen in the flow chart (Figure 1), six studies were eliminated as they were duplicated in the different databases. The studies that remained after the removal of duplicates were screened for inclusion criteria by reading the title and abstract. All studies met the inclusion criteria. Successively, the articles were read out in full text; n = 1 article was excluded because the study sample did not have a baseline physiological state at the pre-intervention measurement phase. A total of six studies were part of this systematic review.

### 3.2. Protocols and Main Study Characteristics

Regarding the general characteristics of the selected studies, they were published between 2016 and 2023 [27,28,29,30,31,32], with the majority (66.67%) published in 2023 [27,28,30,31]. All the studies were carried out in Europe except one that was carried out in New Zealand [29]. Of those carried out in Europe, only one was carried out in Germany (16.67%) [30], while the rest were carried out in Spain (66.67%) [27,28,31,32]. The studies’ typology was heterogeneous, with experimental [30,31,32] and observational studies [27,28,29]; among the experimental studies, there was an open clinical trial [32], a controlled clinical trial [31], and a quasi-experimental study [30]; among the observational studies, there was a cohort study design [29], a descriptive study design [27], and a cross-sectional study design [28]. With regard to the characteristics of the participants, the total number of subjects recruited through the various studies was 148 [27,28,29,30,31,32], the study with the smallest sample size presented 15 subjects (10, 14% of the total) [27], while the study with the largest number of participants had 53 subjects (35.81% of the total) divided into two groups: 27 healthy subjects and 26 with unilateral tendinopathy [28]. The remaining studies comprised between 19 and 22 participants (12.8–14.86% of the total) [29,30,31,32]. The age of the participants ranged from 18 years [27] to 43.12 years [32] with the study by Liu et al. [29] having the lowest mean age 21.10 ± 2.13 and that by Cuevas-Cervera et al. [27] being the study with the highest mean 33.6 ± 10.6. Of the 148 subjects, 44 were female (29.72%) and 104 were male (70.27%). Except for the study by Sanz-López et al. [31], which comprised only men, all other studies formed groups comprising men and women [27,28,29,30,32].

**Figure 2:**
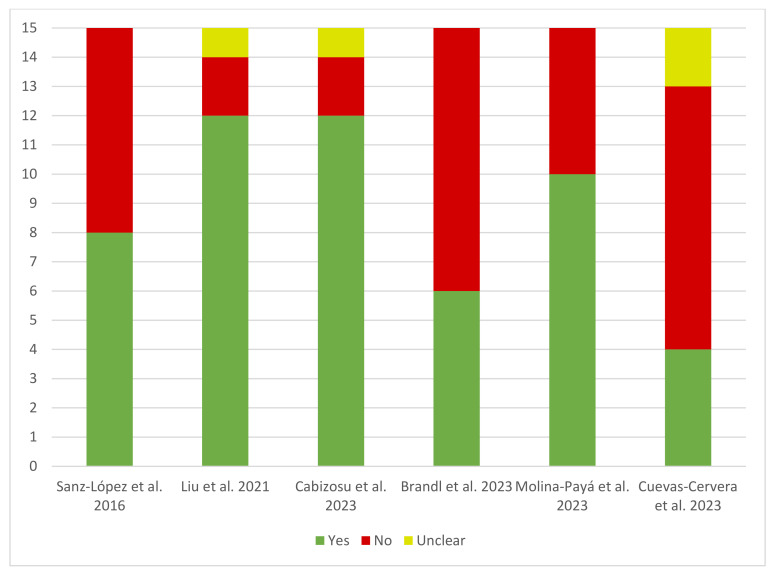
Bar chart of the TISEM scale [24]. Description of the graph: visual quantification of the sum of the points (yes, no, or unclear) for each study [27,28,29,30,31,32].

Regarding the characteristics of the thermographic intervention, the results turn out to be very heterogeneous (Figure 2). Of the recruited studies, the one with the lowest compliance with the checklist items received four “Yes” and a total of nine “No” [32], with the same “No” score obtained by the study of Brandl et al. [30]. The articles with the highest “Yes” score were the studies of Cabizosu et al. [27] and Liu et al. [29] with a score of 12 “Yes”. As for the “No” scores, the studies that received the lowest scores were the studies by Cabizosu et al. [27] and Liu et al. [29] with two “No” results, followed by the article by Molina-Payá et al. [28] with five “No”. As reported above, the studies that received the most “No” were those of Cuevas-Cervera et al. [32] and Brandl et al. [30], with nine “No” while the remaining study received a score of seven “No” [31]. Finally, only three studies scored “Unclear” on any of their items, these being Liu et al. [29] and Cabizosu et al. [27] scoring one “Unclear” and Cuevas-Cervera et al. [32] scoring two.

Regarding the thermographic responses obtained (Table 1), this review highlights heterogeneous information among the various authors about the mean and SD temperature of the patellar region. Calculating the mean value of all the mean temperatures provided by the authors, we obtain a value of 30.78 ± 1 °C [27,28,29,30,31,32], with a lowest mean temperature of 28.2 ± 1.1 °C [30] and the highest of 32.2 ± 1.70 °C [32]. However, it should be noted that the methodological bias in the way temperatures were described was rather heterogeneous in the various articles recruited, as two studies provided an overall mean of the temperature of the two limbs [29,30], two studies described the temperature by classifying the limbs into right and left [27,31], while another study subdivided the limbs into dominant and non-dominant side [32]. Only one study, the only one in which the sample included subjects with pathology (unilateral patellar tendinopathy), subdivided the temperatures of the pathological subjects into healthy and affected side, separating these from the temperatures of the healthy subjects, although the value of the latter is present only as a difference in temperature between limbs and not as an average temperature [28]. The cameras used to obtain the thermograms were FLIR cameras [27,29,30,31,32] except in one study, where an OPTRIS camera was used [28].

### 3.3. Quality of Evidence and Risk of Bias in Included Studies

#### 3.3.1. Quality of Evidence (TISEM Scale)

**Figure 3:**
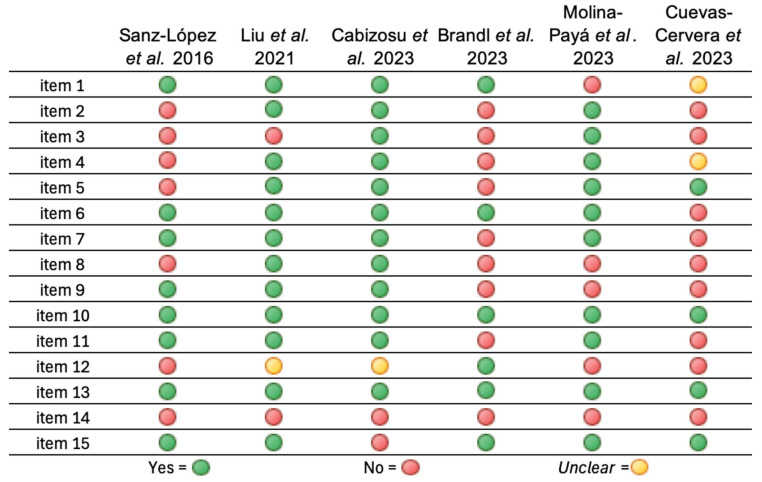
Graphical display of scores for each item of the TISEM scale [24,27,28,29,30,31,32]. Item 1: relevant individual participant data should be provided. Item 2: participants should be instructed to avoid alcoholic beverages, smoking, caffeine, heavy meals, ointments, cosmetics, and showers 4 h before the assessment. In addition, sunbathing (i.e., UV or direct sunbathing without protection) should be avoided before the assessment. Item 3: extrinsic factors that could affect skin temperature (e.g., physical activity prior to assessment, massage, electrotherapy, ultrasound, exposure to heat or cold, cryotherapy) should be clearly provided. Item 4: temperature and humidity of the environment where the assessment has been carried out should be provided as mean ± standard deviation. Item 5: the assessment should be carried out away from any source of infrared radiation (electronic devices, lightning) or draught (under an air-conditioning unit). Item 6: manufacture, model, and accuracy should be provided. Item 7: A period of acclimatization in the assessment room should be carried out. Item 8: if necessary, the chamber should be switched on in advance of the test to allow for the sensors to stabilize along the manufacturers’ guidelines. Item 9: image recording conditions such as average object-to-camera distance and percentage of the region of interest within the image should be detailed. Item 10: the camera should be positioned perpendicular to the region of interest. Item 11: camera emissivity settings should be reported. Item 12: the time of day the images were taken should be reported. Item 13: The standard position of the subject and the region of interest should be well described and appropriately chosen. A visual example (with temperature scale and color scale) is recommended. Item 14: if the skin is dry (e.g., to remove surface water), the drying method should be clearly described. Item 15: the evaluation of thermograms and the collection of software temperatures should be clearly described.

The TISEM scale is responsible for collecting information on the data collection conditions with respect to thermographic measurements. These data are assessed by the inclusion or not of different items when measuring infrared emission, so that the more items included, the less risk of bias in the studies carried out [24]. Of the six articles included, only one study included less than five items, which is why it was classified as a study with low quality of evidence [32]. On the other hand, the three articles that included between 5 and 10 items were classified as medium quality evidence [28,30,31], while two articles described more than 10 items and were classified as high-quality evidence [27,29] (Figure 3).

In an overview, the most described items were perpendicular position of the camera together with description and choice of the standard body position with a visual example, both present in all recruited items [27,28,29,30,31,32]. On the other hand, the least described items were about the skin conditions of the participants (whether the skin was dry and method of drying the skin) and the description of the measurement schedule (Figure 3).

Regarding the demographic information of the sample (Figure 3, items 1–3) the lowest-scoring item was the presence of a description by the authors of extrinsic factors that could have altered skin temperature, of which only 33% of the authors report them, these being Cabizosu et al. [27] and Molina-Payá et al. [28].

Descriptions about relevant participant information were provided by 66.67% of the studies [27,29,30,31]; of the remaining studies, Molina-Payá et al. [28] did not describe relevant participant data and Cuevas-Cervera et al. [32] did not describe them clearly. Pre-measurement instructions were reported by only 50% of the authors, these being the three highest-scoring studies [27,28,29].

Regarding the conditions of the measurement room and the camera configurations used (Figure 3. Items 4–11), the item that was least described was the prior switching on of the chamber for the stabilization of the thermal sensors, which was described by only two authors [27,29], while the item most present in the various studies was the perpendicularity of the chamber with respect to the structure to be assessed; this can be seen in all the studies through the thermograms present in the publications [27,28,29,30,31,32]; two authors also report it in writing in the section on materials and methods [28,29].

The second most described item with respect to the conditions of the measurement room and camera preferences was the description of the information of the cameras used, which was omitted only in the study by Cuevas-Cervera et al. [32]; for the remaining items, there is heterogeneity in the percentage of description by the authors, ranging from 50% in items 4 [27,28,29] and 9 [27,29,31] to 66.67% in items 5 [27,28,29,32], 7 and 11 [27,28,29,31].

Regarding the analysis and reporting of the data (Figure 3, items 12–15), the method of drying the skin was not described by any of the studies included in this review [27,28,29,30,31,32]; as for the measurement schedule, it was only described in the study by Brandl et al. [30]; the description of the schedule in the studies by Liu et al. [29] and Cabizosu et al. [27] was unclear.

The standard position of the subject with a visual example was presented in all studies [27,28,29,30,31,32], while the evaluation of thermograms and the collection of temperatures were described in all but one study [27].

#### 3.3.2. Risk of Bias (QUADAS-2 Scale)

After going through the first three phases of the QUADAS-2 scale [25] with which the systematic review question was assessed, the questions of the scale were adapted to the recruited studies, and any possible bias in study design was ascertained through the flow charts of the various studies.

**Figure 4:**
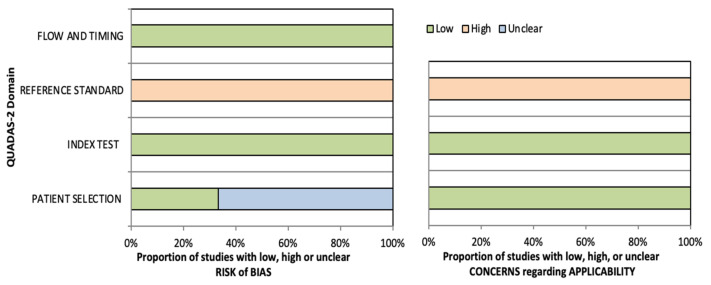
Visual proportioning of results in terms of risk of bias and applicability issues.

We proceeded to submit the various studies recruited to the questions of the four domains provided by the scale, in which the risk of bias was assessed as “low”, “high”, or “unclear”, the same score used to define the problems in terms of applicability [25]. As can be clearly seen in both Table 2 and Figure 4, the results of the various studies were quite homogeneous in almost all domains, both in relation to the risk of bias and the problems of applicability of the diagnostic technique.

The domain that provided the highest “high” for risk of bias was the baseline test domain, in which all studies scored a high risk of bias; the same was observed in the percentage of studies that scored “high” for applicability problems, which was reflected in 100% of the studies [27,28,29,30,31,32]. For the domains of index test and flow and time, all studies scored “low” for risk of bias and scored “low” for applicability problems in the index test domain; the same was observed in the domain of participant selection for applicability problems [27,28,29,30,31,32]. The only domain where heterogeneity between studies could be seen was in the risk of bias in the participant selection domain, where only the studies by Cabizosu et al. [27] and Molina-Payá et al. [28] scored “low” for risk of bias; the remaining studies scored “unclear”.

## 4. Discussion

The aim of this systematic review was to explore all scientific information about thermography and baseline values in the patellar tendon, with the objective of identifying discrepancies in the literature and clarifying the most current knowledge. The results of the literature search provided a total of six studies of different typologies, providing a sample comprising a total of 148 subjects that was then discussed [27,28,29,30,31,32].

Since protocols and regulations for proper thermographic imaging and analysis have been generated, researchers have generated increasingly reliable and reproducible articles. The main results obtained in this review show that the average total temperature obtained in the patellar region was 30.78 ± 1 °C. However, as mentioned above, this value is subject to methodological biases due to substantial differences in thermographic protocols, age, gender, and other sample information.

The various authors provided an overall mean patellar tendon temperature for each limb [27,31,32] or a mean temperature between the two limbs [29,30], except for Molina-Payá et al., who provided only the temperature of the healthy side in the group of subjects with pathology and the temperature difference between limbs in the group of healthy subjects [28]. However, a correct subdivision of temperatures according to individual sample data such as sex, body mass index (BMI), and age should have been described since, among other intrinsic factors of the subjects, these individual traits give the subjects very different characteristics in terms of infrared radiation emission. This is due to the difference in the capacity for thermal dispersion and consequent capacity for heat transfer through radiation [33,34,35,36,37,38,39,40], thus justifying that future research should be directed towards the classification and subdivision of thermal ranges according to sex, BMI, and age. Moreover, considering the great influence of the muscular system on the tendon metabolic activity, it would be interesting to consider the possible influence of the tone and strength of the quadriceps muscle on the metabolic activity of the patellar tendon.

Ferreira et al. provided a pre-exercise temperature difference of the lower extremities in a group of young (mean age 22.3 ± 2.2) and elderly (mean age 66.8 ± 5.1) individuals, highlighting a higher temperature in the young group with statistically significant differences between groups [36]; however, the skin temperature difference at older ages should not have interfered with the results of the sample of this review, as the age range is quite limited and homogeneous in most studies.

In addition, other authors compare temperatures between genders in different age ranges providing similar conclusions that women have a lower skin temperature than men in many body areas [33,39,40,41]; the main cause of this heterogeneity was not so clear. In fact, different authors studied the existence of a relationship between temperature and the percentage of subcutaneous fat in women or the percentage of muscle mass in men [34,35,37,39]. In 2015, Neves et al. detected statistically significant differences in the involvement of the thickness of the subcutaneous fat layer in affecting skin temperature at rest [38]. Furthermore, it is known that authors with samples like those in the studies included in this review in terms of age and health conditions obtained homogeneous thermographic results, which fall within the range of temperatures obtained by the studies analyzed in this review, although the thermographic test was performed after a 5 min warm-up [42].

It is known from other authors that thorough and detailed pre-imaging instructions regarding activities, beverages, and leisure are necessary so that the skin temperature is not altered at the time of the test [43,44,45,46]. In this regard, only half of the authors reported having provided prior instructions to the subjects [27,28,29]. The general tendency in the literature is to use only some of the instructions [36,47], while other authors such as Fernandes et al. described them as exclusion criteria [48]. However, Molina-Paya et al. did not provide data about the physical activity performed by the subjects [28], while Cuevas-Cervera et al. did not describe them clearly [32]. The importance of this depends on the fact that according to some authors, alcohol, tobacco, or coffee can alter basal metabolism, so data about the intake or consumption of these elements prior to the thermographic protocol should be recorded [44,45].

Something similar to what was detected for previous instructions was observed regarding the description of the use of specific physiotherapeutic techniques or exposure to physical agents, such as electrotherapy, cryotherapy, or massages [49]. Although only Cabizosu et al. and Molina-Payá et al. provided this information in their studies [27,28], a heterogeneous trend was found in providing this information with authors describing them [33] and others not [17,50]. It is known, from other authors, that there are physiotherapy techniques that can generate vasoconstrictor or vasodilator processes that alter the thermographic images up to 48 h after they have been performed [49]; so, in order to provide reliable values, it is extremely important to collect this information.

Regarding the environment in which the thermographic imaging was developed, there are very important aspects that should be taken into account to obtain reliable data; among these, we can find the humidity and temperature of the environment [51]; half of the authors correctly reported these data [27,28,29], while one author did not describe them clearly [32].

More than 40 years ago, authors like Salisbury et al. described humidity and temperature by providing mean values with their SDs [52], as well as authors like Fernandes et al. having described them in more recent times [48]; however, in the literature, different ways of providing these data were found. For example, some authors provided them by means of ranges [53]. The importance of taking these data into account depends on the fact that at different temperatures and humidities, thermoregulatory processes are generated that can provide altered thermographic patterns. Processes such as sweating or convection are dependent on the atmospheric environment and can generate abnormal thermographic patterns due to associated physiological responses [16,51,54,55,56,57,58].

Regarding the equipment and protocols, with the exception of Brandl et al. [30] and Sanz et al. [31], all the other studies recruited performed the tests far from sources of infrared radiation or under air current; compliance with this condition was necessary so that there would be no alteration in skin temperature [24]. It is known from other authors that not respecting this precondition could lead to biases in the collection of thermographic data due to alterations in the uptake by the thermographic camera [24]. In addition, Ring et al. [58] described the possibility of having to switch on the thermal imaging camera beforehand to stabilize the sensors and provide a more correct image. The TISEM protocol recommends that a pre-test should be carried out in accordance with the manufacturers’ guidelines [22].

Only two studies of those recruited in this systematic review described turning on the cameras in advance [27,29]. Regarding camera-to-subject distance and region of interest (ROI), Mazdenyasta et al. investigated the role of subject-to-camera distance and concluded that it should depend on camera resolution and subject size [51]; regarding ROI, in another publication, Molina-Payá et al. [59] discussed the importance of ROI for inter- and intra-professional test reproducibility while Selfe et al. [60] described the points to be taken into account for marking ROI in the patellar region. Of the recruited studies, only half described the ROI and the distance between the thermographic camera and the subject; Cuevas-Cervera et al. [32] did not describe the ROI, while Brandl et al. [30] and Molina-Payá et al. [28] did not describe the distance between camera and subject.

Considering all the above, it is clear that there is a need for studies of thermographic reference values with a much more reliable and adjusted protocol and homogeneity of the sample. Detecting skin temperature reflecting alterations in the underlying tissues could be of great help in the assessment of metabolic response in sick participant, so future studies should present a larger sample of participants, create a subdivision of temperatures by factors intrinsic to the participant such as age, sex, and BMI; and provide data as exhaustive as possible about the measurement protocol and the study environment. In addition, due to the close relationship between the appearance of tendon injuries and the functioning of the muscular system, future studies could evaluate the physiological and metabolic response of tendons after the application of specific muscle activation protocols. This could help physiotherapists and sports science graduates in the management of tendinopathies and in the evaluation of applied treatments.

## 5. Study Limitations

The main limitation we encountered was the scarcity of quantitative and qualitative information about thermographic reference values in the patellar tendon. This is probably because thermography is not currently a main clinical diagnostic tool, so there are large differences in protocols, samples, and measurement techniques by each author, which makes it difficult to compare and pool the results. Furthermore, as there is no scale for risk of bias assessment in diagnostic studies that can cover all typologies of study designs, the QUADAS-2 scale had to be adjusted for the recruited articles. This meant that the questions in some domains had to be modified in order to make sense of them, a process that is bibliographically endorsed by the creators of the scale [25]. In this specific case, the QUADAS-2 scale does not correspond perfectly to its main function because it was adapted for experimental studies; however, it remains the best tool for assessing the risk of bias in diagnostic studies [61].

## 6. Conclusions

The mean value found in the patellar region was 30.78 ± 1 °C, although due to the evident lack of homogeneity in the protocols executed and the samples found in the studies, this value should be verified in future studies. Moreover, only two studies had sufficiently high methodological quality to support these data; thus, a future study of patellar tendon reference values in healthy individuals following the most current recommendations and protocols is necessary. Although a general thermal value for patellar tendon has been proposed in this review, future research is needed to confirm or refute it.

## Figures and Tables

**Table 1:** Data extraction from the included studies.

Study Characteristics						Sample Characteristics			Thermographic Characteristics	Results Obteined	
Autor	Year	Study Type	Sample Country	Publication Country	Journal	Sample Size	M. Age	Sex	TISEM Scale	ΔT (°C)	Termograph
Cabizosu et al. [27]	2023	Descriptive	Spain	Switzerland	*Sensor*	G1: 15	18–30	M: 11	YES: 1; NO: 3; UN:1	R 31.68 ± 1.4	Flir E75 (Wilsonville, USA)
								W: 4		L 31.71 ± 1.2	
Molina-Payá et al. [28]	2023	Transversal	Spain	USA	*Sage Journals*	G1: 26 (UT)	G1 = 30.1 ± 9.0	M: 33	YES: 10; NO: 5; UN: 0	G1 healthy side: 31.10 ± 1.66	OPTRIS PI 450 IRT (Berlin, Germany)
						G2: 27	G2 = 23.3 ± 5.38	W: 20		G2: n/m	
Liu et al. [29]	2021	Cohort	New Zealand	Netherlands	*Physical Therapy in Sport*	G1: 18	21.10 ± 2.13	M: 12	YES: 12; NO: 2; UN: 1	30.13 ± 1.51	FLIR T450SC (Wilsonville, USA)
								W: 6			
Brandl et al. [30]	2023	Quasi-experimental	Germany	USA	*PLOS ONE*	G1: 21	18–60	M: 15	YES: 6; NO: 9; UN: 0	28.2 ± 1.1	Flir One (Wilsonville, USA)
Sanz-López et al. [31]	2016	Controlled clinical trial	Spain	USA	*Journal of Strength and Conditioning Research*	G CONT: 10	22.72 ± 4.21	M: 19	YES: 8; NO: 7; UN: 0	R = CONT 29.87 ± 0.75, EXC 30.92 ± 0.82.	FLIR Thermacam E60 (Boston, USA)
						G EXC: 9		W: 0		L = CONT 29.86 ± 0.71, EXC 30.83 ± 0.85	
Cuevas-Cervera et al. [32]	2023	Open clinical trial	Spain	Switzerland	*Diagnostics*	G1: 22	33.6 ± 10.6	M: 14	YES: 4; NO: 9; UN: 2	LD: 32.1 ± 1.47	X FLIR T420bx
								W: 8		LND: 32.2 ± 1.70	

ΔT: mean temperature; CONT: control; DS: dominant side; EXC: eccentric exercise; G: group; L: left; M: men; M. age: mean age; NDS: non-dominant side; N/m: no mention; R: right; UN: unclear; USA: United States of America; UT: unilateral tendinopathy; W: women.

**Table 2:** Summary of risk of bias and applicability issues: author judgement on each domain of the studies included.

	Risk of Bias				Applicability Concerns		
	Patient Selection	Index Test	Reference Standard	Flow and Timing	Patient Selection	Index Test	Reference Standard
Cabizosu et al. 2023 [27]	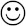	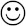	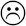	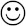	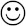	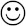	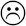
Molina-Payá et al. 2023 [28]	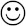	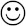	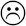	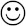	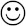	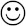	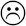
Liu et al. 2021 [29]	?	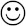	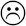	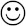	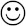	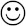	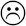
Brandl et al. 2023 [30]	?	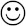	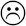	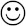	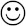	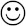	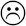
Sanz-López et al. 2016 [31]	?	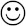	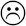	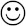	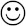	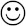	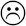
Cuevas-Cervera et al. 2023 [32]	?	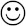	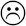	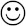	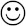	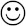	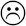

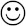
 Low; 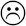 High; ? Unclear.

## Data Availability

Data generated or analyzed during this study are included in this article.
